# Orphan Nuclear Receptor Nur77 Regulates Androgen Receptor Gene Expression in Mouse Ovary

**DOI:** 10.1371/journal.pone.0039950

**Published:** 2012-06-28

**Authors:** Anyi Dai, Guijun Yan, Qinyuan He, Yue Jiang, Qun Zhang, Ting Fang, Lijun Ding, Jianxin Sun, Haixiang Sun, Yali Hu

**Affiliations:** 1 Reproductive Medicine Center, The Affiliated Drum Tower Hospital of Nanjing University Medical School, Nanjing, People's Republic of China; 2 Center for Translational Medicine, Thomas Jefferson University, Philadelphia, Pennsylvania, United States of America; Florida International University, United States of America

## Abstract

The androgen receptor (AR) is a nuclear receptor that is expressed in growing follicles and involved in folliculogenesis and follicle growth. The orphan nuclear receptor, Nur77, also has an important role in steroid signaling and follicle maturation. We hypothesized that AR levels and androgen signaling through AR are regulated by Nur77 in the ovary. In the ovaries of *Nur77* knockout mice (n = 5), real-time PCR results showed that the mRNA levels of *AR* and an androgen signaling target gene, *Kitl*, were decreased by 35% and 24%, respectively, relative to wild-type mice (n = 5), which suggested transcriptional regulation of AR by Nur77 *in vivo*. In cultured mouse granulosa cells and a steroidogenic human ovarian granulosa-like tumor cell line, KGN, mRNA and protein expression levels of AR were increased by overexpressing Nur77 but decreased by knocking down endogenous Nur77. Consistent with increased AR expression, chromatin immunoprecipitation showed that Nur77 bound to the NGFI-B response element (NBRE) in the *AR* promoter sequence. *AR* promoter activity was stimulated by Nur77 in HEK293T cells and attenuated in Nur77 knockout mouse granulosa cells (luciferase assay). Overexpression of Nur77 enhanced the androgenic induction of Kitl (200 nM; 48h), while knockout of *Nur77* attenuated this induction. These results demonstrate that AR is regulated by Nur77 in the ovaries, and they suggest that the participation of Nur77 in androgen signaling may be essential for normal follicular development.

## Introduction

Recently, the study of female reproductive physiology has been focused on the genesis and development of ovarian follicles. Follicular development and function are controlled by a plethora of intrinsic and endocrine factors, and are affected by interactions between multiple cell types within the ovaries [1].

The Nur77 [nerve growth factor induced-B (NGFI-B), nuclear receptor 4A1 (NR4A1)] orphan nuclear receptor is a transcription factor that activates target genes [2]. Nur77 has been shown to recognize a specific binding site called the NGFI-B response element (NBRE; AAAGGTCA), which is present in a subset of genes [3], [4], and Nur77 has been shown to have an effect on transcriptional activation. Nur77 is predominantly expressed in theca cells and granulosa cells of ovarian follicles [5]. Nur77 has a variety of functions, including promoting expression of genes involved in the endocrine, nerve, and immune systems [6]. In the endocrine system, Nur77 has been shown to participate in the regulation of the following genes related to the production of steroidogenic enzymes: *Pomc* [7] and *Cyp17a1* [8] in rats; *StAR* [9] and *Cyp21a1* [3] in mice; and *CRH* [10] and *HSD3B2* [11] in humans.

The androgenic steroid hormones are mostly produced by the adrenal glands and gonads of both sexes. Although androgens are present at much lower concentrations in the circulation of females relative to males, they are essential in maintaining female physiological functions, especially intraovarian functions, such as stimulating follicular growth on early stages [12], enhancing granulosa cell apoptosis [13], and regulating the function and lifespan of the corpus luteum [14]. Androgen signal transduction requires the androgen receptor (AR), which is a member of the nuclear receptor family that has been found to be expressed in growing follicles [15]. In an *AR*-deficient mouse model, there is an age-dependent progression in ovarian abnormality with a premature ovary failure (POF) phenotype that includes lower follicle numbers, impaired mammary development, fewer pups per litter, and a marked increase of atretic follicles, which indicates that AR-mediated androgen signaling is indispensable for the maintenance of folliculogenesis, and that impaired androgen signaling is a potential cause of the POF syndrome [16]. Nevertheless, the direct involvement of androgen and AR in ovarian follicles is still not well-known.

Nur77 and AR are both involved in follicle maturation. Nur77 is expressed in corpora lutea and cultured ovarian granulosa cells, and it activates steroidogenic gene expression during luteinization, thus, indicating a functional relevance to stage-specific expression during follicular maturation [6]. AR is expressed in all cell types of an ovarian follicle, including oocytes, granulosa cells, and theca cells [17]. Androgen is capable of triggering oocyte maturation in *Xenopus laevis* [18], mouse [19], and pig [20] most likely via the classical AR mechanism. In cultured granulosa cells or follicles of various species *in vitro*, AR mediates the stimulatory effects of androgen on proliferation and follicular development [12], [21], [22]. However, the mechanism of AR regulation is unknown.

Therefore, this study aimed at investigating a novel role for Nur77 in AR regulation by testing the hypothesis that AR expression and signaling are affected by Nur77. The mRNA expression patterns of *AR* and *Kitl* in the ovaries were examined in *Nur77* knockout and wild-type mice to determine the regulation of AR by Nur77 *in vivo*. The mRNA and protein levels of AR and Kitl were analyzed in cultured mouse granulosa cells and a steroidogenic human ovarian granulosa-like tumor cell line, KGN, after overexpression and knockdown of Nur77 *in vitro*. The interactions between AR and Nur77 were also studied using chromatin immunoprecipitation and *AR* promoter activity. Moreover, we showed that Nur77 regulated Kitl expression through stimulation or inhibition of AR signaling using androgen or antiandrogen flutamide, thus, demonstrating an important interaction between Nur77 and androgen signaling in the ovaries.

## Materials and Methods

### Animals

*Nur77* ^+/−^ mice (B6.129S2-*Nr4a1^tm1Jmi^*/J) were purchased from Jackson Laboratory (Bar Harbor, Maine, USA). Three-week-old ICR mice were purchased from the Lab Animal Center of Yangzhou University (Yangzhou, China). All animals were maintained in the Animal Laboratory Center of Drum Tower Hospital (Nanjing, China) on a 12∶12-h light/dark cycle (lights off at 19∶00) with food and water available ad libitum. All animal experiments were performed according to the guidelines of the Experimental Animals Management Committee (Jiangsu Province, China).

### Cell lines

The KGN cell line (a generous gift from Dr. Yiming Mu at the General Hospital of the People's Liberation Army, Beijing, China), which was established from a human GCT and expresses typical granulosa cell markers, was cultured as previously described [23]. HEK293T cells and HEK293A cells were maintained in DMEM/F12 (Gibco BRL/Invitrogen, Carlsbad, CA, USA) supplemented with 10% fetal bovine serum (FBS; HyClone, Thermo Scientific, South Logan, UT, USA), and antibiotics (100 IU/ml penicillin and 100 µg/ml streptomycin; Gibco BRL/Invitrogen) at 37°C in a humidified environment with 5% CO_2_.

### Collection and culture of mouse primary granulosa cells (mGCs)

Mouse granulosa cells were collected from the ovaries of immature mice (21 day old) as previously described [24]. In brief, the ovaries were harvested and cleared from the surrounding fat. The ovaries were then punctured with 25 gauge needles, and the cells were collected into phenol red-free DMEM/F12 (Gibco BRL/Invitrogen) containing 0.2% BSA, 10mM HEPES, and 6.8mM EGTA. The cells were incubated for 15 min at 37°C under 95% O_2_:5% CO_2_ and centrifuged for 5 min at 250×g. The pellets were suspended in DMEM/F12 containing 10% fetal bovine serum, 1 mM pyruvic acid (Gibco BRL/Invitrogen), 2 mM glutamine and antibiotics (100 IU/ml penicillin, and 100 µg/ml streptomycin). The oocytes were filtered out with a 40-µm cell strainer. The mouse granulosa cells were cultured in the suspended medium at 37°C in a humidified environment with 5% CO_2_, and they were used within three passages in this study.

### Construction of recombinant adenovirus

Adenoviruses harboring the full-length Nur77 gene (Ad-Flag-Nur77) and adenoviruses harboring human Nur77 siRNA oligonucleotides (5′-AAGUUGUCCGAACAGACAGCCUGA-3′; Ad-siNur77) were generated using the AdMax (Microbix Biosystems, Inc., Toronto, Canada) and pSilencer™ adeno 1.0-CMV (Ambion, Austin, TX, USA) systems as previously described [25]. Adenovirus bearing LacZ (Ad-LacZ) was obtained from BD Biosciences Clontech (Palo Alto, CA, USA). Viruses were packaged and amplified in HEK293A cells and purified using CsCl banding followed by dialysis against 10 mmol/L Tris-buffered saline with 10% glycerol. Titering was performed on HEK293A cells using the Adeno-X Rapid Titer Kit (BD Biosciences Clontech) according to the manufacturer's instructions.

### RNA isolation and quantitative real-time PCR

Total RNA was extracted from tissues and cultured cells using the TRIzol reagent (Invitrogen, Carlsbad, CA, USA). cDNA was synthesized from 1mg of purified total RNA using a PrimeScript RT reagent kit (Bio-Rad Laboratories, Hercules, CA, USA) according to the manufacturer's instructions. The specific primers used for quantitative polymerase chain reaction (PCR) analysis are listed in Table 1. Each real-time PCR reaction had the following components: 1 µl of RT product, 10 µl of SYBR Green PCR Master Mix (Bio-Rad Laboratories), and 500 nM forward and reverse primers. Real-time PCR was performed on a MyiQ Single Color Real-time PCR Detection System (Bio-Rad Laboratories) for 40 cycles (95°C for 10 sec; 60°C for 30 min) after an initial 3 min incubation at 95°C. Each sample was analyzed in triplicate, and the experiment was repeated three times. Data were analyzed using the 2^−△△CT^ method [26], and the fold change in expression of each gene was normalized to the endogenous control (18S rRNA).

**Table 1:** Oligonucleotide primer sequences for quantitative real-time PCR.

Species	Genes	Primers (5′–3′)	Products size (bp)
Mouse	*AR*	GGACCATGTTTTACCCATCG	171
		TCGTTTCTGCTGGCACATAG	
Mouse	*Kitl*	TTGTTACCTTCGCACAGTGGCTGGT	166
		AGCGAAGCACTCTGCTCCAACAA	
Mouse	*Nur77*	GCACAGCTTGGGTGTTGATG	187
		CAGACGTGACAGGCAGCTG	
Mouse	*18S rRNA*	ATGGCCGTTCTTAGTTGGTG	183
		CGGACATCTAAGGGCATCAC	
Human	*AR*	CCTGGCTTCCGCAACTTACAC	168
		GGACTTGTGCATGCGGTACTCA	
Human	*KITLG*	TGGTGGCAAATCTTCCAAAAG	76
		CAATGACTTGGCAAAACATCCA	
Human	*Nur77*	ACCCACTTCTCCACACCTTG	200
		ACTTGGCGTTTTTCTGCACT	
Human	*18S rRNA*	CGGCTACCACATCCAAGGAA	186
		CTGGAATTACCGCGGCT	

### Western blot analysis

The proteins were prepared and separated on SDS-PAGE as previously described [27]. Cells were rinsed twice with ice-cold PBS (pH 7.4) and lysed with lysis buffer (50 mM Tris-HCl, pH 7.6; 150 mM NaCl; and 1.0% NP-40) containing protease inhibitor cocktail (Sigma, St. Louis, MO, USA). The protein concentrations were measured by the Bradford assay (Bio-Rad Laboratories). Equal amounts of total protein (40 ug) were separated on a 10% SDS-polyacrylamide gel and transferred onto polyvinylidene fluoride membrane (Millipore, Billerica, MA, USA). Immunoblotting was performed with primary antibodies against hAR (1∶500; Bioworld Technology Inc., Minneapolis, MN, USA), Nur77 (1∶1000; Cell Signaling Technology, Danvers, MA, USA), and mAR (1∶500; Santa Cruz Biotechnology, Inc., Santa Cruz, CA, USA). β-actin (1∶5000; Abcam, Cambridge, MA, USA) was measured as an internal control. Immunodetection was accomplished using a goat anti-rabbit (1∶5000; Bio-Rad Laboratories) or goat anti-mouse (1∶10000; Bio-Rad Laboratories) secondary antibody, and an enhanced chemiluminescence detection kit (Millipore).

### Transient transfection and luciferase reporter assays

Approximately 383 bp of the mouse *AR* promoter sequence (−2509/−2127) was amplified by PCR from mouse granulosa cell genomic DNA with the following primers: 5′-TATAGGTACCGGCCTCAACTGCCTTACTCACAGC-3′ and 5′-TAGCAAGCTTTGGCGTTGGTGGAGTATGCAATCA-3′. Approximately 1.2 kb of the human *AR* promoter sequence (−2837/−1638) was amplified by PCR from human granulosa-luteinized (hGL) cell genomic DNA with the following primers: 5′-TATAGGTACCATCATTGGTTGCCTGAGGAG-3′ and 5′-TAGCAGATCTGGCAGGATGGTAGAATGGAA-3′. The PCR products were cloned into the pGL3-basic luciferase reporter plasmid (Promega, Madison, WI, USA) and sequenced to confirm the resulting plasmid. Preconﬂuent (75–80%) HEK293T cells in 12-well plates were transfected with pCMV-Flag-Nur77 or pCMV-empty vector (pCMV-EV), and the AR firefly luciferase report plasmid using Nanofectin (PAA, Pasching, Austria). mGCs (70% confluent) from mice with different genotypes were transfected with the AR luciferase report plasmid using Lipofectamine 2000 (Invitrogen). All cells were cotransfected with the Renilla luciferase reporter plasmid (pRL-RSV; Promega) as a control for transfection efficiency. Luciferase activity was assayed 48 h after transfection using the Luciferase Assay System (Promega) and measured with a Centro XS3 LB 960 luminescence counter (Berthold Technologies, GmbH Co., KG, Germany). At least three transfection assays were performed to obtain statistically significant data.

### Chromatin immunoprecipitation (ChIP) assay

ChIP was performed based on the protocol provided in the kit with some modifications (ChIP assay kit; Upstate Biotechnology, Lake Placid, NY, USA) as previously described [28]. Briefly, mGCs or KGN cells (70–80% confluent) were infected with Ad-LacZ and Ad-Flag-Nur77 (10 MOI) for 48 h then washed with PBS and crosslinked with 1% formaldehyde for 15 min at room temperature. Cross-linking was stopped with the addition of glycine (0.125 M final concentration) for 10 min. The cells were washed twice with cold PBS and harvested in lysis buffer A (20 mM Tris-HCl, pH 8.0; 85 mM KCl; 1 mM EDTA; 0.5 mM EGTA; and 0.5% Nonidet P40) containing protease inhibitor cocktail (Sigma), and the cells were then pelleted by centrifugation. The cell pellets were lysed in nuclear lysis buffer B (50 mM Tris-HCl, pH 8.0; 10 mM EDTA; and 1% SDS) containing protease inhibitor cocktail. The cell samples were sonicated in ice to yield genomic DNA fragments with sizes of approximately 500–1000 bp. Then, the precleared supernatants were incubated with 30 µl of anti-FLAG M2 affinity agarose gel (Sigma) and rotated at 4°C overnight. The immune complexes were pulled down by protein A/G beads (Upstate Biotechnology) and eluted by incubation at 65°C for 30 min followed by an incubation at room temperature for 15 min in fresh elution buffer (1% SDS and 0.1 M NaHCO_3_). The crosslinks were reversed by incubation at 65°C for 5 h with a final concentration of 0.3M NaCl. DNA was purified by phenol: chloroform extraction and ethanol precipitation after the eluates were incubated with proteinase K. Finally, the purified DNA fragments were used as templates for PCR amplification and products were visualized on agarose gels. The specific primers were as follows: 5′-TATAGGTACCGGCCTCAACTGCCTTACTCACAGC-3′ and 5′-TAGCAAGCTTTGGCGTTGGTGGAGTATGCAATCA-3′ (spanning −2509/−2127 bp) for mouse *AR* promoter DNA fragments and 5′-TATAGGTACCATCATTGGTTGCCTGAGGAG-3′ and 5′-TAGCAGATCTGGCAGGATGGTAGAATGGAA-3′ (spanning −2837/−1638 bp) for human *AR* promoter DNA fragments.

To provide stronger evidence about Nur77 binding to NBRE in human AR promoter, independent quantitative ChIP experiments were performed. For these studies, chromatin was co-immunoprecipitated from KGN lysates with an antibody to Nur77 or irrelevant antibody (goat anti-rabbit IgG) as control. Lysates were sonicated to generate chromatin fragments of 200 bp which were amplified by quantitative real time PCR. The sets of primers included: 5′-AGTGCTCCAGGTGGAAGAGA-3′ and 5′-CTCTGGAAAGCCAGGATGAG-3′ (spanning −2553/−2401 bp), 5′-AGGAAAGAGTGGAGGGAGGA-3′ and 5′-AGGATGAGCTGACCTTTGGA-3′ (spanning −2584/−2413 bp) which both overlap putative Nur77-binding site of human *AR* promoter; 5′-TCCTTAAATGCAGGGTCCAC-3′ and 5′-AAATGCCCTGTGATTTCAGC-3′ (spanning −4713/−4526 bp), 5′-GAGTCGTTGGAGGACCTGAA-3′ and 5′-CCTGTCACTGTCTCCCCATT-3′ (spanning −4119/−3967 bp) as negative control region.

### Avidin-biotin conjugate DNA (ABCD) precipitation assay

Double-stranded oligonucleotides were designed for ABCD assays and biotinylated at the 3′-end of the sense strand. The following primers were designed: 5′ biotin–CACCATTTTCCAAAGGTCAGCTCATCCTGG-3′ for human AR wild-type F (from −2440 to −2411 bp); and 5′ biotin–CACCATTTTCCAgAatTCAGCTCATCCTGG-3′ for human AR mutant F. KGN cells infected with Ad-LacZ and Ad-Flag-Nur77 (10MOI) for 48 h were lysed with lysis buffer (10 mM Tris–Cl, pH 7.8; 1 mM EDTA; 150 mM NaCl; and 0.1% Nonidet P40) containing protease inhibitor cocktail at 4°C. The cell extracts were incubated with 500 pmol of each double-stranded DNA immobilized on streptavidin agarose beads in binding buffer (10 mM Tris, pH 8.0; 150 mM NaCl; 0.5% TritonX-100; 0.5 mM DTT; 0.5 mM EDTA; 10% glycerol; and 20 μg/ml poly [dI–dC]) containing protease inhibitor cocktail for 4 h at 4°C. The protein complexes on the beads were separated by SDS-PAGE, transferred onto polyvinylidine fluoride membrane and probed with an anti-Flag-HRP antibody (1∶3000; Sigma). Immunodetection was performed using an enhanced chemiluminescence detection kit.

### Statistical analysis

Data were expressed as the means ± SEM from at least three independent experiments. Student's *t*-test was performed for comparison between the mean values for two groups, and ANOVA was used to detect differences among more than two groups Values were determined to be significant when *P*<0.05.

## Results

### *AR* and *Kitl* mRNA levels decrease in Nur77^−/−^ mice ovaries

**Figure 1:**
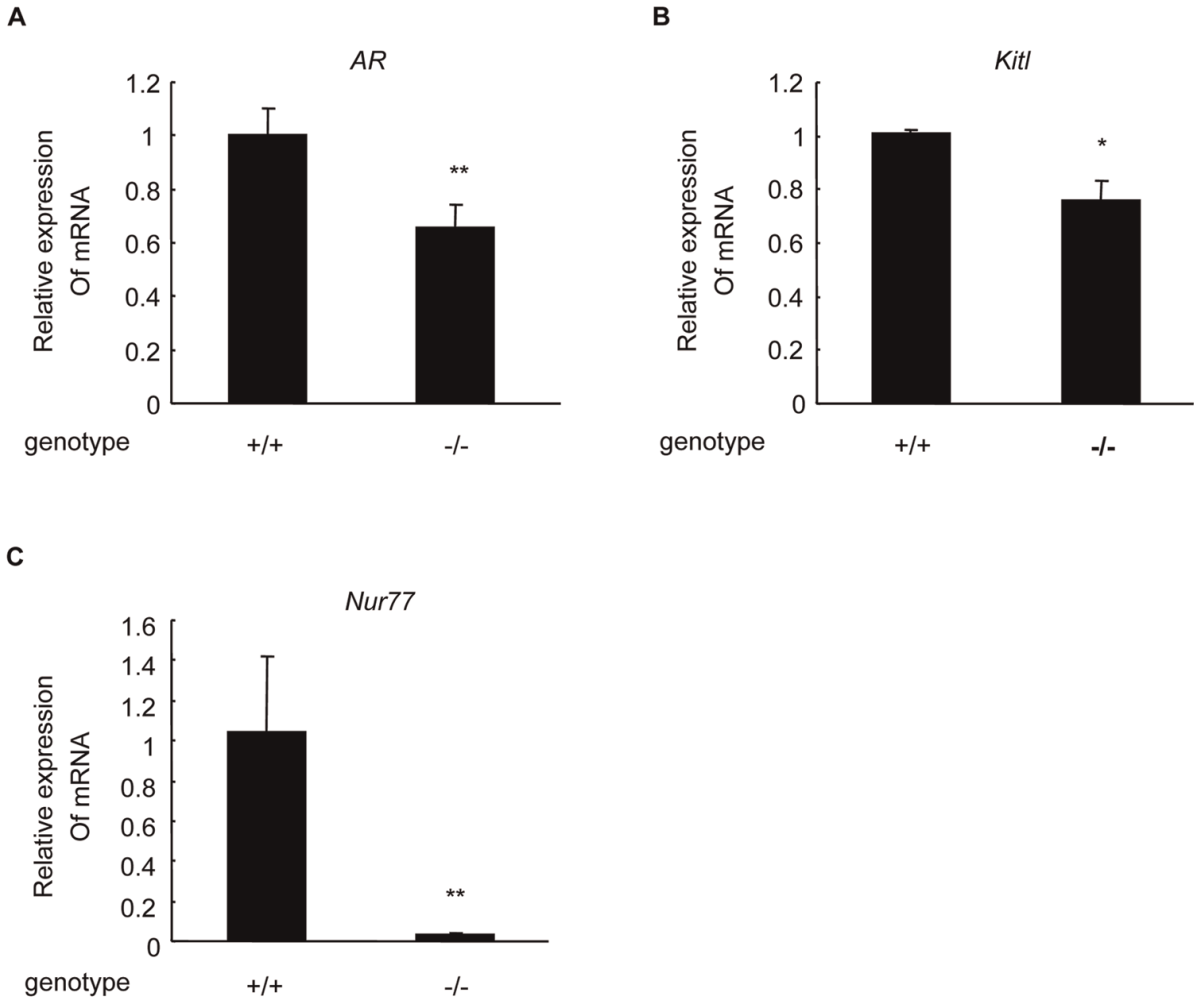
AR and Kitl mRNA levels decreased in *Nur77* ^−/−^ mouse ovaries. RNA was collected from 20-week-old *Nur77* ^+/+^ and *Nur77* ^−/−^ female mouse ovaries and reverse transcribed for real-time PCR. *AR* (**A**), *Kitl* (**B**) and *Nur77* (**C**) mRNA levels of *Nur77* ^−/−^ mouse ovaries (n = 5) versus *Nur77* ^+/+^ mouse ovaries (n = 5) were measured and expressed as relative quantities (*P<0.05, **P<0.01).

To determine if AR and Kitl in the ovaries were regulated by Nur77 *in vivo*, *AR* and *Kitl* mRNA expression levels were analyzed with real-time PCR in the ovaries from *Nur77* ^+/+^ and *Nur77* ^−/−^ 20-week-old female mice. In comparison with *Nur77* ^+/+^ ovaries, the expression levels of *AR* mRNA (Figure 1A) and *Kitl* mRNA (Figure 1B) were decreased by 35% and 24%, respectively, in *Nur77* ^−/−^ ovaries (Figure 1C).

### Nur77 increases AR and Kitl expression in mouse primary granulosa cells

**Figure 2:**
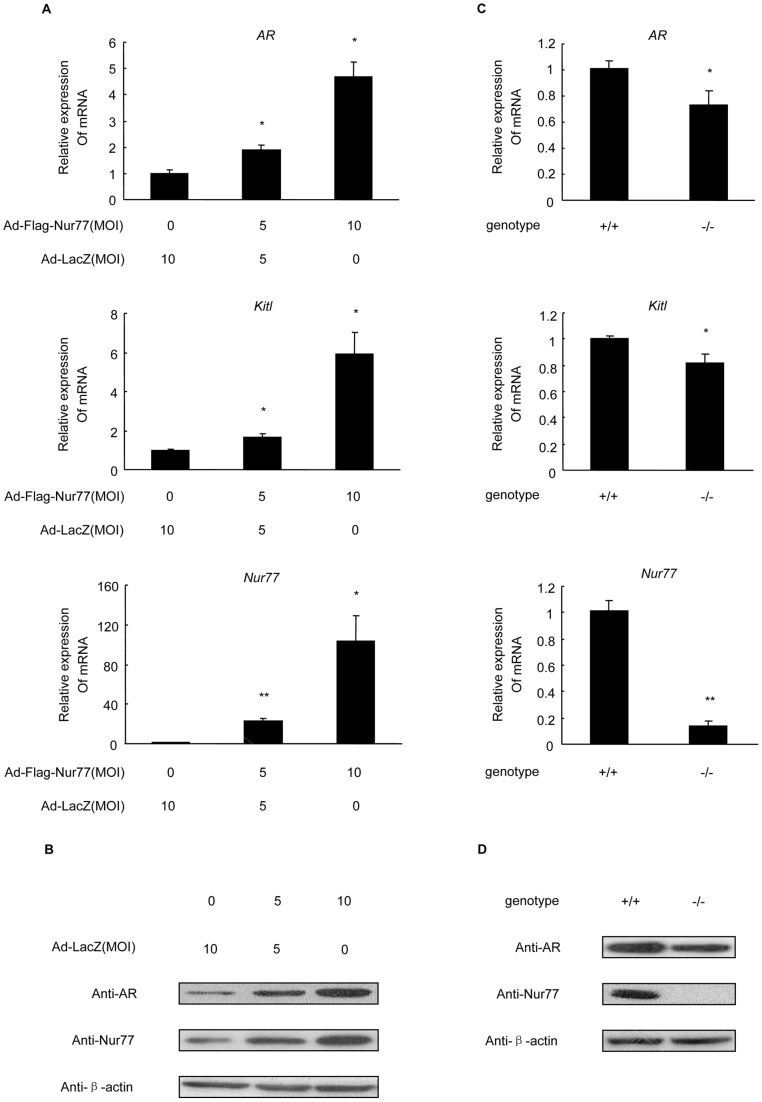
Effects of Nur77 overexpression and knockout on mouse AR and Kitl expression. A: *Nur77* ^+/+^ mGCs were infected with Ad-LacZ or Ad-Flag-Nur77 at the indicated MOI for 48 h. AR and Kitl mRNA levels were measured by real-time PCR and shown as a ratio over control (Ad-LacZ). **B:** AR protein levels were measured by western blot analysis. **C:** Real-time PCR analysis of AR and Kitl mRNA expression in *Nur77* ^+/+^ and *Nur77* ^−/−^ mGCs from 3-week-old mice. **D:** Western blot analysis of AR protein expression in Nur77^+/+^ and Nur77^−/−^ mGCs from 3-week-old mice. The results are an average of three independent experiments performed in triplicate (*P<0.05, **P<0.01).

To investigate the functional role of Nur77 in the regulation of AR and Kitl expression in mGCs, we used adenoviral technology to alter the endogenous Nur77 protein expression in *Nur77* ^+/+^ mGCs at an MOI of 0, 5 and 10 for 48 h, and we collected RNA for real-time PCR analysis and protein for western blot analysis. We found that adenovirus-mediated overexpression of Nur77 in *Nur77* ^+/+^ mGCs increased both AR mRNA and protein expression levels (Figure 2A, B) in a concentration-dependent manner. Moreover, as shown in Figure 2A, Kitl mRNA expression was significantly increased by approximately 6.0-fold when Nur77 was overexpressed at an MOI of 10 in *Nur77* ^+/+^ mGCs.

To further confirm the regulatory effect of Nur77 on AR and Kitl expression *in vivo*, we isolated mGCs from *Nur77* ^+/+^ and *Nur77* ^−/−^ female mouse ovaries. As expected, both AR and Kitl mRNA expression levels were significantly decreased in *Nur77* ^−/−^ mGCs as compared to wild-type mGCs (Figure 2C). Furthermore, AR protein expression decreased (Figure 2D) in purified mGCs from *Nur77* ^−/−^ female mouse ovaries. Together, these findings strongly suggested that Nur77 is involved in the regulation of AR and Kitl expression in mouse ovary granulosa cells.

### Nur77 binds to the mouse *AR* promoter and stimulates its activity

**Figure 3:**
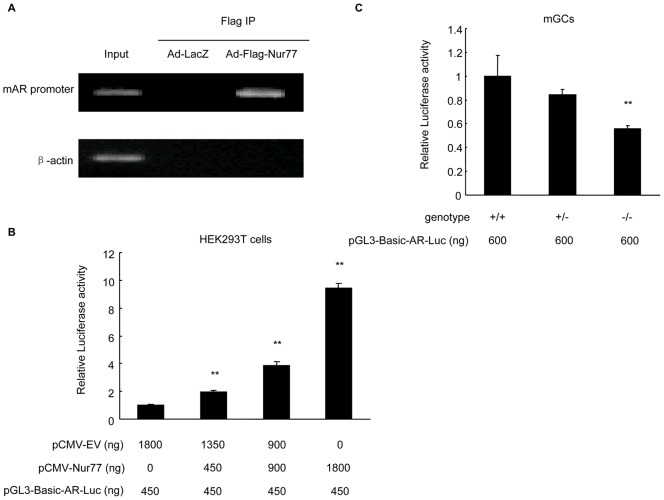
Nur77 binds to the mouse *AR* promoter and stimulates its activity. **A:** The results of ChIP-PCR amplification are shown using primers against the mouse *AR* promoter region. PCR products were separated by agarose gel electrophoresis. **B:** HEK293T cells were transfected with pCMV-Nur77 or pCMV-empty vector plasmid and cotransfected with the m*AR* promoter-luciferase construct using the Nanofectin transfection reagent. After 48 h, luciferase assays were performed and normalized by constitutive Renilla luciferase. **C:** mGCs from *Nur77* ^+/+^, *Nur77* ^+/−^ and *Nur77* ^−/−^ mice were transfected with m*AR* promoter-luciferase construct using Lipofectamine 2000 reagent. After 48 h, luciferase assays were performed and normalized by constitutive Renilla luciferase (n = 3; *P<0.05, **P<0.01).

Nur77 has been reported to regulate target gene expression through direct binding to the NBRE sequence (AAAGGTCA) as monomers (4) and/or to the palindromic NurBE sequence as homodimers (7). Based on the mouse *AR* promoter sequence deposited in the Transcriptional Regulatory Element Database (accession No. 83828), we found a potential NBRE (TCAGGTCA) within m*AR* promoter core regions. To examine if Nur77 can bind to the *AR* promoter *in vivo*, we performed a ChIP-PCR assay. Confluent mGCs were infected with Ad-LacZ and Ad-Flag-Nur77 at an MOI of 10 for 48 h. Soluble chromatin from the mGCs was then immunoprecipitated with an anti-FLAG M2 affinity agarose gel, and the purified DNA fragments from the Flag-Nur77-Chromatin complexes were analyzed by PCR with the specific primer pairs designed for the NBRE region of the m*AR* promoter. Figure 3A showed that the *AR* promoter was efficiently recovered from the Flag-Nur77 immunoprecipitates but not from the control immunoprecipitates (Ad-LacZ), suggesting that Nur77 directly binds to the chromatin-associated *AR* promoter in mouse granulosa cells.

To test if Nurr77 directly regulates m*AR* promoter activity, we measured *AR* gene promoter activity in HEK293T cells and mGCs by transient transfection and luciferase reporter assays. As shown in Figure 3B, overexpression of Nur77 in HEK293T cells enhanced m*AR* promoter activity in a concentration-dependent manner. In mGCs from *Nur77* ^−/−^ mice, however, the m*AR* promoter activity was significantly lower than that in mGCs from *Nur77* ^+/+^ mice (Figure 3C). Together, these data suggested that Nur77 binds to the m*AR* promoter and increases AR promoter activity.

### Nur77 up-regulates AR and KITLG expression levels in human granulosa cells

**Figure 4:**
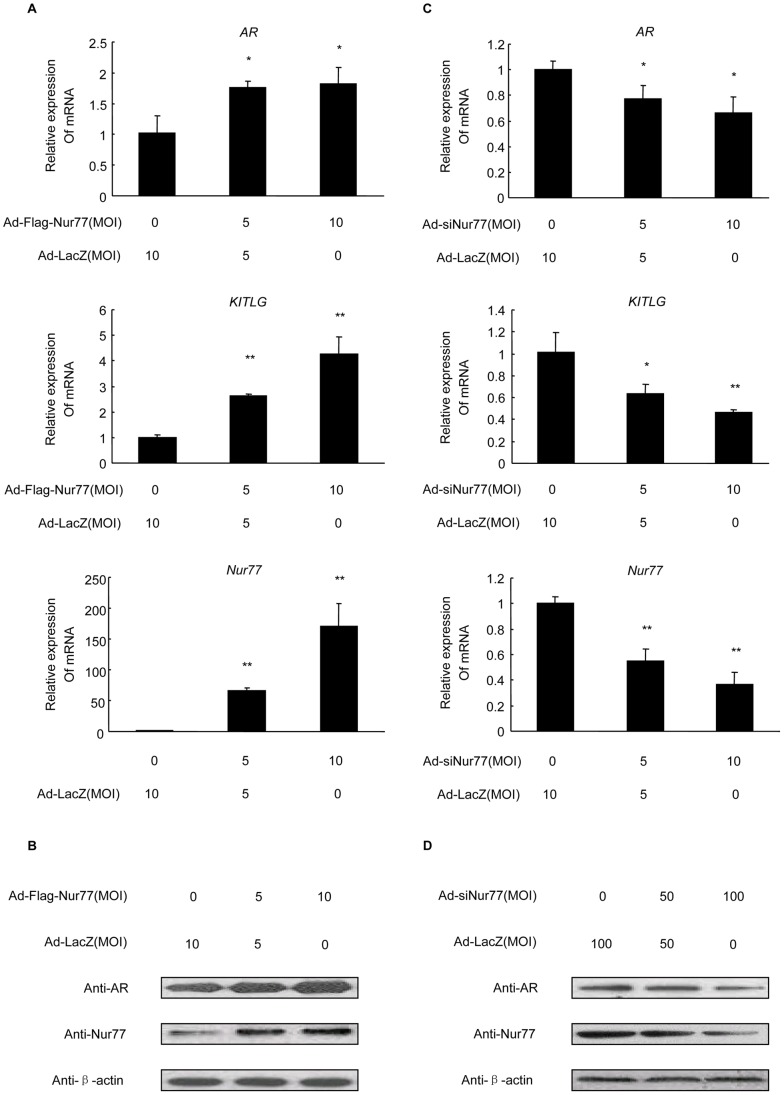
Effects of Nur77 overexpression and silencing on human AR and KITLG expression in KGN cells. A: KGN cells were infected with Ad-LacZ or Ad-Flag-Nur77 at the indicated MOI for 48 h, and AR and KITLG mRNA levels were measured by real-time PCR and shown as a ratio over control (Ad-LacZ). (n = 3; *P<0.05, **P<0.01). **B:** Western blot analysis of AR protein expression in KGN cells treated with Ad-LacZ or Ad-Flag-Nur77 at the indicted MOI for 48 h. **A:** Real-time PCR analysis of AR and KITLG mRNA expression levels in KGN cells infected with Ad-LacZ or Ad-siNur77 (MOI = 50 and 100) for 48 h (n = 3; *P<0.05, **P<0.01). **B:** Western blot analysis of AR protein expression in KGN cells treated with Ad-LacZ or Ad-siNur77 at the indicated MOI for 48h.

To assess if Nur77 also regulates the transcription of the human AR gene, we treated KGN cells, a human granulosa-like tumor cell line, with Ad-Flag-Nur77 at the indicated MOI for 48 h. Using real-time PCR analysis, we found that mRNA levels of hAR and hKITLG were dramatically elevated in a dose-dependent manner with increasing expression levels of Nur77 (Figure 4A). Moreover, western blot analysis indicated an increase in AR protein levels an increase in Figure 4B).

To further support the role of Nur77 in modulating hAR expression, we performed loss of function studies using an RNA interference technique. KGN cells were infected with Ad-siNur77 at MOIs of 0, 50 or 100, and total RNA and protein were measured by real-time PCR and western blot analysis, respectively. As expected, Ad-siNur77-infected cells showed a substantial reduction in endogenous Nur77 mRNA expression. The treatment subsequently inhibited AR and KITLG mRNA expression levels. AR mRNA was down-regulated by the inhibition of endogenous Nur77 to 54%, and KITLG expression was repressed to 42% (Figure 4C). AR protein expression was also attenuated accompanied by repression of Nur77 (Figure 4D). Thus, these results suggest that Nur77 promotes hAR expression in KGN cells.

### Nur77 binds to the human *AR* promoter and enhances its activity

**Figure 5:**
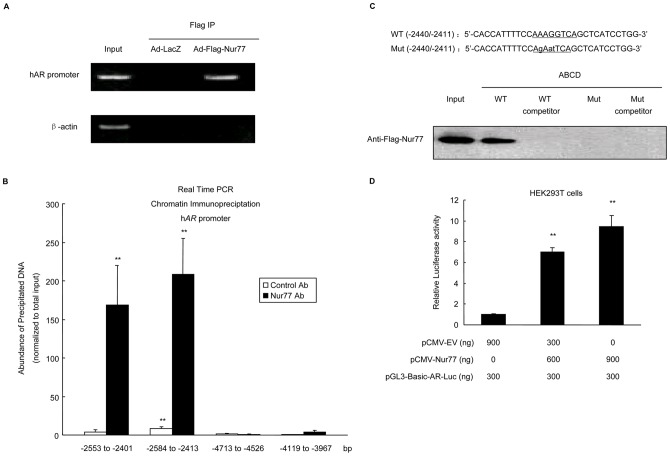
Nur77 binds to the human *AR* promoter and enhances its activity. **A:** The results of ChIP-PCR amplification are shown using primers against the human *AR* promoter region. PCR products were separated by agarose gel electrophoresis. **B:** Quantitative ChIP was performed with an antibody to Nur77 or irrelevant control antibody. Co-precipitating chromatin fragments were analyzed by real time PCR using primer sets that span −2553/−2401 bp, −2584/−2413 bp, −4713/−4526 bp and −4119/−3967 bp in human *AR* promoter region. Results were normalized to total input (not precipitated) chromatin(n = 3; *P<0.05, **P<0.01). **C:** ABCD assays were performed using biotinylated or unbiotinylated (competitor) double-stranded AR wild-type (WT) and AR mutant (Mut) oligonucleotides in addition to KGN cell extracts after infection with Ad-LacZ or Ad-Flag-Nur77. **D:** HEK293T cells were transfected with pCMV-Nur77 or pCMV-empty vector and cotransfected with the h*AR* promoter-luciferase construct using the Nanofectin transfection reagent. Forty-eight hours after transfection, luciferase assays were performed and normalized by constitutive Renilla luciferase (n = 3; *P<0.05, **P<0.01).

In addition, as the h*AR* promoter sequence has consensus AAAGGTCA binding sites located at −2429 to −2422 relative to the start site of AR transcription (Transcriptional Regulatory Element Database; accession No. 43390), we first conducted a ChIP-PCR assay as described in mGCs above, and similar results were obtained (Figure 5A). We also used quantitative chromatin immunoprecipitation to further determine if Nur77 binds to the conservative NBRE within the h*AR* promoter core region *in vivo*. For these studies, cell lysates were sonicated to generate ∼200bp chromatin fragments prior to immunoprecipitation with an antibody to Nur77 or irrelevant control antibody. Co-precipitating chromatin was amplified by real-time PCR using primers spanning the putative Nur77-binding site (−2429 to −2422bp). Primers used in these experiments were designed to generate a product of <200bp. It was found that chromatin fragments with potential NBRE, instead of negative control region, specifically co-precipitated with Nur77 from lysates of KGN cells (Figure 5B).

We next investigated if Nur77 interacts with the human *AR* promoter *in vitro*. ABCD assays were performed using biotin conjugate, double-stranded oligonucleotides containing the sequences shown in Figure 5C. The whole cell lysates extracted from intact KGN cells infected with Ad-Flag-Nur77 or Ad-LacZ at an MOI of 10 were mixed with oligonucleotides and immobilized on streptavidin agarose beads. Nur77 strongly bound to the AR wild-type oligonucleotides but not to the mutant sequence (Figure 5C), thus, suggesting that this NBRE site interacts with Nur77 in a sequence-specific manner.

To further verify the direct transcriptional effect of Nur77 on AR in KGN cells, we generated a construct corresponding to approximately 1.2 kb of the human *AR* promoter sequence (−2837/−1638) in a luciferase reporter plasmid and transiently transfected HEK293T cells to measure *AR* gene promoter activity. Overexpression of Nur77 activated h*AR* promoter activity in a concentration-dependent manner (Figure 5D). These data demonstrated that the conserved NBRE (−2429 to −2422) is required for Nur77 specific binding and functional activation of human *AR* promoter activity.

### Nur77 affects the expression of the androgen signaling target gene, Kitl, by regulating AR in granulosa cells

**Figure 6:**
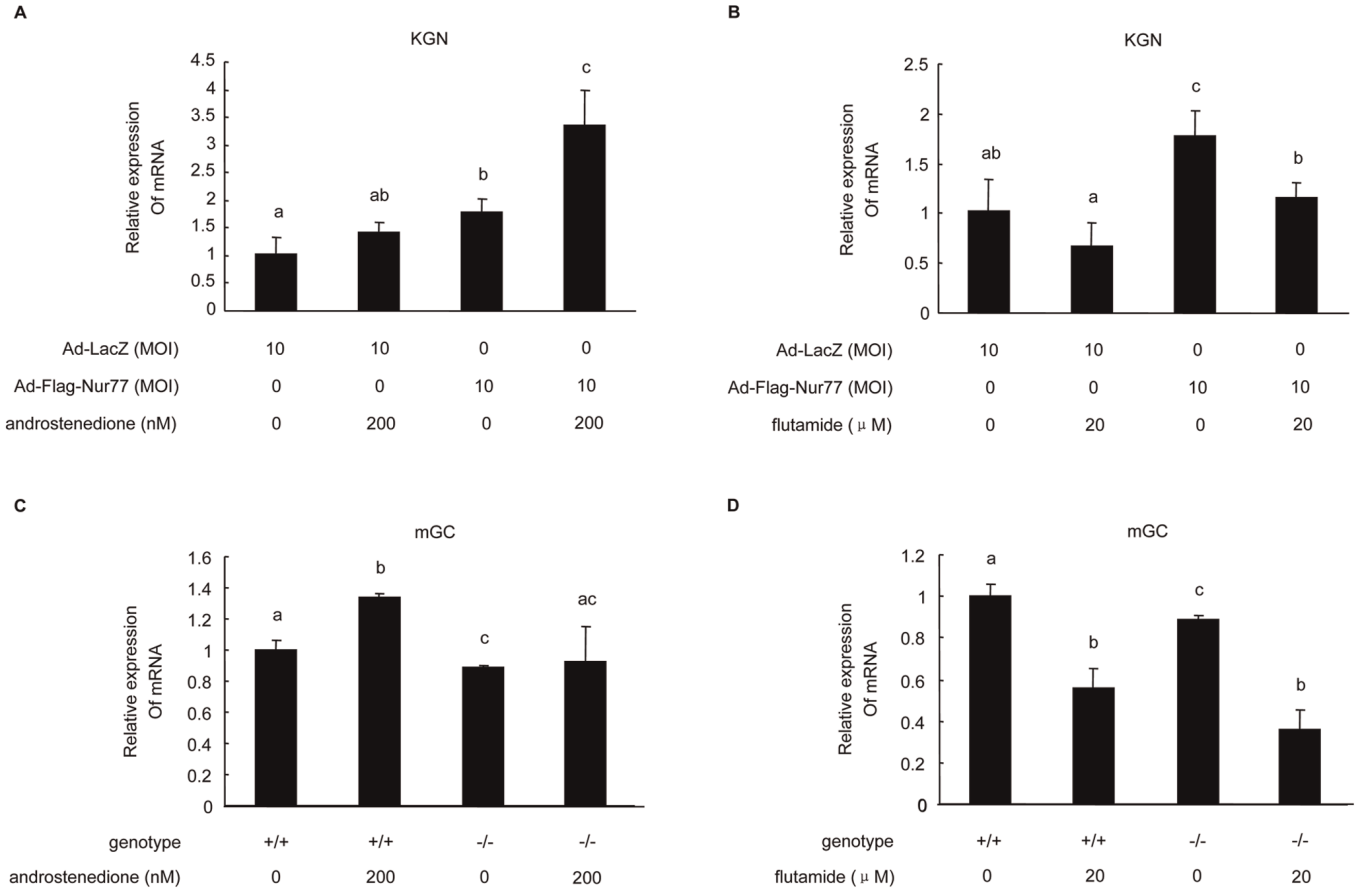
Nur77 affected the expression of the androgen signaling target gene, *Kitl*, by regulating AR. KGN cells were infected with Ad-LacZ or Ad-Flag-Nur77 at 10 MOI, and *Nur77* ^+/+^ or *Nur77* ^−/−^ mGCs were treated with androstenedione (**A, C**) or the androgen signaling antagonist, flutamide (**B, D**). Cellular RNA was collected, and real-time PCR analysis showed Kitl expression levels with different treatments. The results are shown as a ratio over control (n = 3). Values with different superscripts (a, b, c, d) are significantly different (p<0.05) from each other.

To investigate the mechanism of Kitl gene regulation by Nur77 and AR, KGN cells infected with Ad-LacZ or Ad-Flag-Nur77 at an MOI of 10 and mGCs isolated from *Nur77* ^+/+^ and *Nur77* ^−/−^ female mouse ovaries were treated with androstenedione (AD) or an androgen signaling antagonist, flutamide (FL). Real-time PCR analysis showed that Kitl expression was elevated by AD in KGN cells, which is consistent with a previous report [16]. FL treatments, however, markly attenuated Kitl expression in KGN and *Nur77* ^+/+^ mGCs (Figure 6). The stimulatory effects of AD were intensified in KGN cells overexpressing Nur77 (Figure 6A). In *Nur77* ^−/−^ mGCs, the positive effect of AD was almost completely inhibited (Figure 6C) while the inhibitory effect of FL was enhanced (Figure 6D). Taken together, these findings strongly suggest that Nur77 generates its physiological function in granulosa cells partly through up-regulating of AR expression.

## Discussion

Previous studies have shown that Nur77 was originally identified by its rapid activation by nerve growth factor in PC12 cells [29] and by serum growth factors in fibroblasts [30]. Nur77 belongs to the NR4A family of orphan nuclear receptors along with two other members, NURR1 (NR4A2) and NOR1 (NR4A3) [2]. These family members possess similar structural features with a conserved DNA binding domain and a ligand binding domain, but these members have a variable sequence in the N-terminal AF-1 domain [3]. These receptors act as endpoint effectors in the protein kinase A (PKA) signaling pathway. The AF-1 domain of Nur77 has a key role in transcriptional activation, intramolecular interactions, intermolecular interactions, and cofactor recruitment [31], [32]. Nur77 is expressed in various systems, including ovary, testis, adrenal gland, muscle, thymus, pituitary, and central nervous system [6]. Nur77 is known to be regulated in the following ways: by luteinizing hormone in testicular Leydig cells [33]; by follicle-stimulating hormone in mouse testis tissue and human fetal Sertoli cells [34]; and by gonadotropin during follicle development in rat ovaries [8]. Nur77 regulates the expression of genes, such as *Cyp21*, *Cyp11b2*, *HSD3B2* and *StAR*, encoding steroidogenic enzymes in the adrenal glands, testes and ovaries [35]–[37], and all of these genes are involved in androgen biosynthesis, thus, suggesting that Nur77 may be important in androgen production as well as its function through AR. Furthermore, both Nur77 and AR are particularly relevant to ovarian follicular maturation [6], [18]–[20]. In the present study, we found that Nur77 positively regulates AR expression in components of the female ovaries. Different granulosa cells, such as mGCs and KGN cells, were used in this study to allow the evaluation and confirmation of the interactions between Nur77 and AR.

The luciferase assays suggested a direct role in transcriptional regulation of AR expression by Nur77. Because a conserved NBRE element (AAAGGTCA) and a partial NBRE sequence (AGGTCA) have been observed in human and mouse *AR* promoters respectively, further experiments, such as ChIP and ABCD assays were conducted in the current study. These assays suggested that Nur77 was recruited to the *AR* promoter and that Nur77 bound to the NBRE to mediate *AR* promoter activation.

As the other two members of the NR4A family, Nurr1 and Nor1, have similar expression patterns and transcriptional roles as Nur77 [38]. It was proposed that there might be potential functional redundancy between NR4A family members [39], [40]. However in the thymuses of Nur77^−/−^ mice, Nurr1 expression was proved to be faint and not increased [41]. In our study, Nurr1 level in the ovary of Nur77^−/−^ mice seemed to have a fluctuation along with the mouse age (data not shown). In addition, expression levels of Nurr1 and Nor1 in human ovarian follicles and corpora lutea are much lower than Nur77 [11]. Thus they may not have a maximal role in regulating AR as Nur77 in the ovaries.

Kit ligand (Kitl) is a growth factor that influences target cells through binding to its tyrosine kinase receptor, Kit [42]. The granulosa cell-derived Kitl can bind to the Kit receptor expressed in oocytes [43], thus, having an important role in the following functions: primordial follicle activation [44]; oocyte growth and survival [45]; meiotic maturation of preovulatory follicles [46]; granulosa cell proliferation [47]; and regulation of ovarian steroidogenesis [48]. Antiandrogen flutamide attenuates Kitl induction by 5α-dihydrotestosterone in mouse ovaries and KGN cells. Moreover, androgen-induced transactivation of mouse and human *KITLG* promoters has been observed using a luciferase reporter assay in KGN, 293T, and HeLa cells [16]. These studied have established that Kitl represents a direct downstream target of androgen signaling in a regulatory cascade controlling folliculogenesis. In the current study, we found that the changes in Kitl occurred along with changes in AR after the alterations in Nur77 both *in vivo* and *in vitro*. To ensure this effect was androgen-dependent, we performed the Nur77 overexpression experiments in KGN cells and *Nur77^+/+^*mGCs maintained in complete medium (DMEM/F12 medium plus 10% FBS), phenol-free medium (phenol-free medium plus 10% FBS), or androgen-free medium (phenol-free medium with charcoal/dextran-treated FBS). The results provided evidence that Kitl was regulated by Nur77 in the presence of exogenous androgen, whereas its expression was not up-regulated in androgen-free circumstance (Figure S1, S2). Therefore we consider that Nur77, as an upstream regulator, may indirectly increase androgen-induced Kitl expression through the stimulation of AR, thus, contributing to oocyte growth and maturation.

Global *AR* knockout (ARKO) female mice are subfertile, and they have defective folliculogenesis. Moreover, ARKO female mice ultimately develop premature ovarian failure [16], [49]. AR signaling in granulosa cells regulates female fertility possibly by controlling preantral follicle growth and development to antral follicles and preventing follicular atresia [50]. Consistent with the function of AR stimulation mediated by Nur77, we postulate that Nur77 represents one of the primary molecules participating in female fertility and folliculogenesis. As there was only the capacity of reproduction which had been analyzed in Nur77^−/−^ mice as a measure of reproductive function in previous studies [39], [41], additional detailed studies need to be performed in our future research.

In conclusion, AR protein expression is positively regulated by Nur77 at least at the transcriptional level through the AR promoter in mouse and human granulosa cells. AR signaling is also mediated by Nur77 as shown by changes in Kitl expression. The transcriptional down-regulation is also confirmed in the ovaries from *Nur77* knockout mice *in vivo*. Our study provides new insights into Nur77 functions in the female reproductive system. Understanding the crosstalk between Nur77 and AR signaling helps us to understand the mechanisms of androgen signaling, follicle growth and oocyte maturation, which are the key pathological processes in diseases, such as premature ovarian failure (POF) and polycystic ovary syndrome (PCOS; hyperandrogenic anovulation).

## Supporting Information

Figure S1 **Effects of Nur77 overexpression on mouse AR and Kitl expression.** *Nur77* ^+/+^ mGCs were infected with Ad-LacZ or Ad-Flag-Nur77 at the indicated MOI for 48 h. Cells were cultured in complete medium (**A,** DMEM/F12 medium plus 10% FBS), phenol-free medium (**B,** phenol-free medium plus 10% FBS), or androgen-free medium (**C,** phenol-free medium with charcoal/dextran-treated FBS). AR and Kitl mRNA levels were measured by real-time PCR and shown as a ratio over control (Ad-LacZ). The results are an average of three independent experiments performed in triplicate (*P<0.05, **P<0.01).
(TIF)

Figure S2 **Effects of Nur77 overexpression on human AR and KITLG expression.** KGN cells were infected with Ad-LacZ or Ad-Flag-Nur77 at the indicated MOI for 48 h. Cells were cultured in complete medium (**A,** DMEM/F12 medium plus 10% FBS), phenol-free medium (**B,** phenol-free medium plus 10% FBS), or androgen-free medium (**C,** phenol-free medium with charcoal/dextran-treated FBS). AR and KITLG mRNA levels were measured by real-time PCR and shown as a ratio over control (Ad-LacZ). The results are an average of three independent experiments performed in triplicate (*P<0.05, **P<0.01).
(TIF)
