# Supplementary figures and images for: Orphan Nuclear Receptor Nur77 Regulates Androgen Receptor Gene Expression in Mouse Ovary
Source: PLoS One. 2012 Jun 28;7(6):e39950. doi: 10.1371/journal.pone.0039950 (PMC3386274; doi:10.1371/journal.pone.0039950)

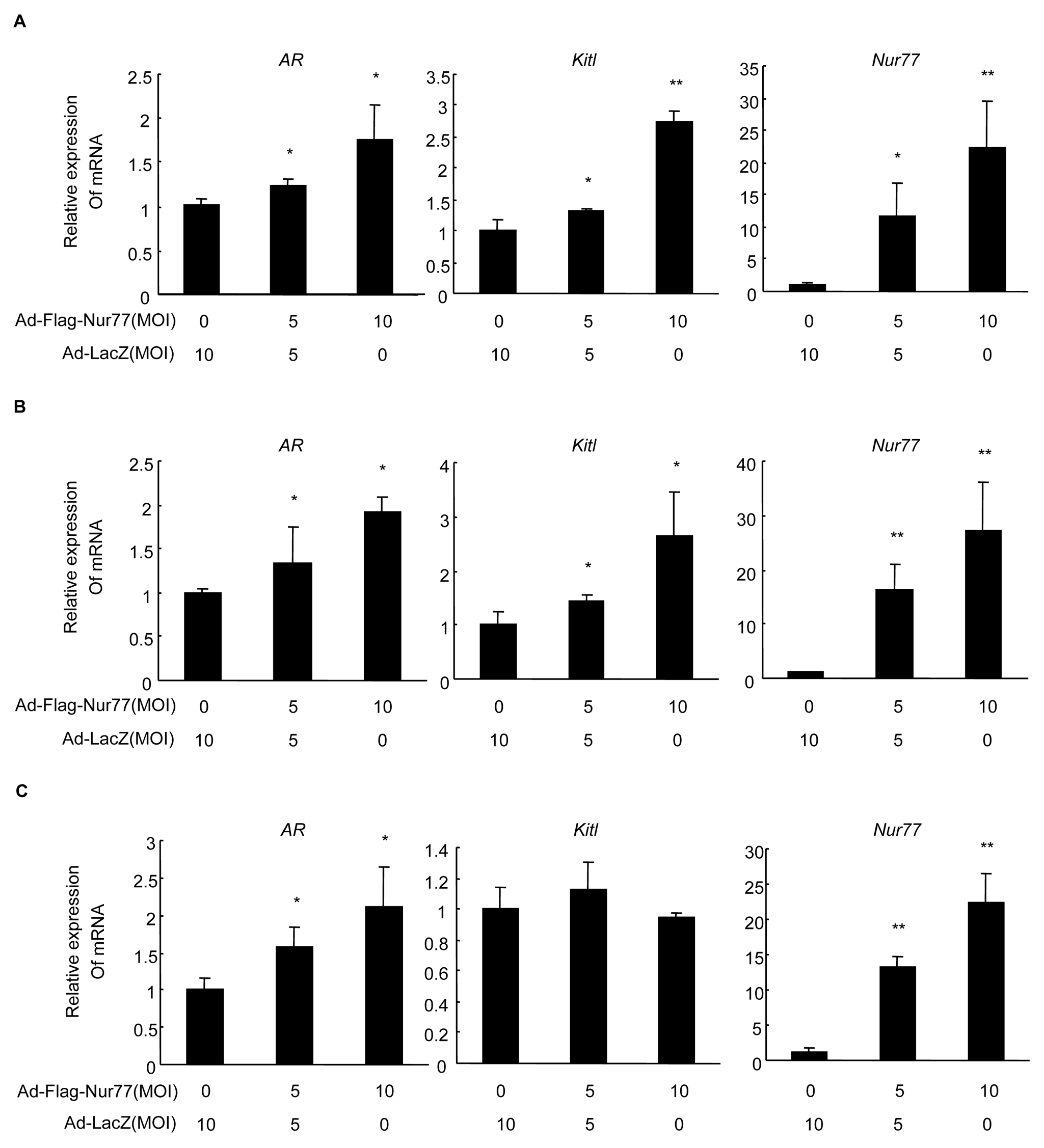

Supplement: Figure S1 — Effects of Nur77 overexpression on mouse AR and Kitl expression. Nur77 +/+ mGCs were infected with Ad-LacZ or Ad-Flag-Nur77 at the indicated MOI for 48 h. Cells were cultured in complete medium (A, DMEM/F12 medium plus 10% FBS), phenol-free medium (B, phenol-free medium plus 10% FBS), or androgen-free medium (C, phenol-free medium with charcoal/dextran-treated FBS). AR and Kitl mRNA levels were measured by real-time PCR and shown as a ratio over control (Ad-LacZ). The results are an average of three independent experiments performed in triplicate (*P<0.05, **P<0.01). (TIF) [file pone.0039950.s001.tif]

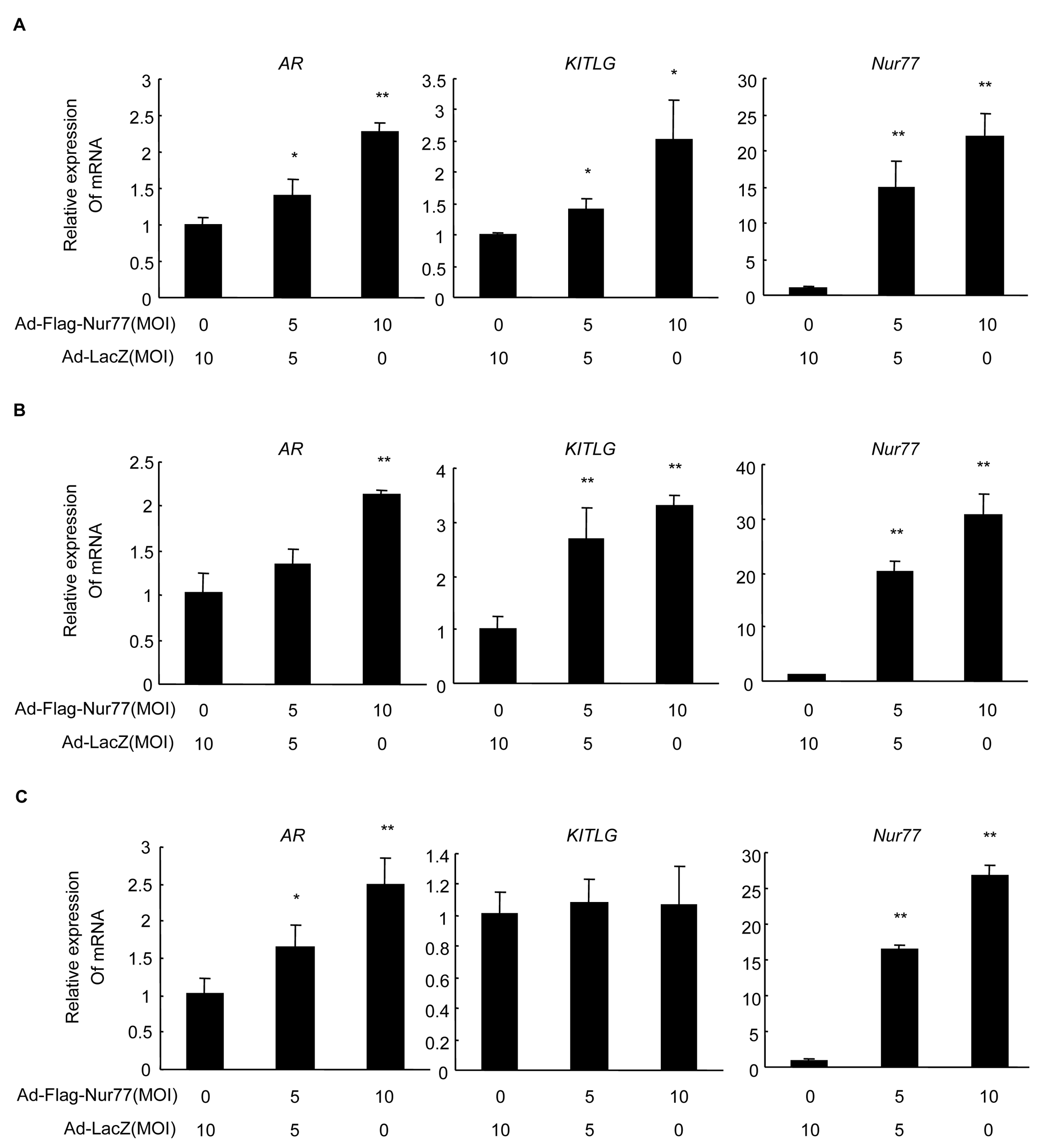

Supplement: Figure S2 — Effects of Nur77 overexpression on human AR and KITLG expression. KGN cells were infected with Ad-LacZ or Ad-Flag-Nur77 at the indicated MOI for 48 h. Cells were cultured in complete medium (A, DMEM/F12 medium plus 10% FBS), phenol-free medium (B, phenol-free medium plus 10% FBS), or androgen-free medium (C, phenol-free medium with charcoal/dextran-treated FBS). AR and KITLG mRNA levels were measured by real-time PCR and shown as a ratio over control (Ad-LacZ). The results are an average of three independent experiments performed in triplicate (*P<0.05, **P<0.01). (TIF) [file pone.0039950.s002.tif]
